# Anastomosing hemangioma simulating renal cell carcinoma

**DOI:** 10.1590/S1677-5538.IBJU.2016.0653

**Published:** 2017

**Authors:** Mariana Athaniel Silva Rodrigues, Eduardo Kaiser Ururahy Nunes Fonseca, Fernando Ide Yamauchi, Ronaldo Hueb Baroni

**Affiliations:** 1 Departamento de Imagem, Hospital Israelita Albert Einstein, São Paulo, SP, Brasil

**Keywords:** Radiology, Kidney, Magnetic Resonance Imaging

## Abstract

The anastomosing hemangioma is a recent described rare variant, which histologically simulates an angiosarcoma and occurs primarily in the genitourinary tract. We present a case of renal anastomosing hemangioma from a radiologic perspective, describing its imaging features and reviewing its presentation and management.

## CASE PRESENTATION

**Figure 1:**
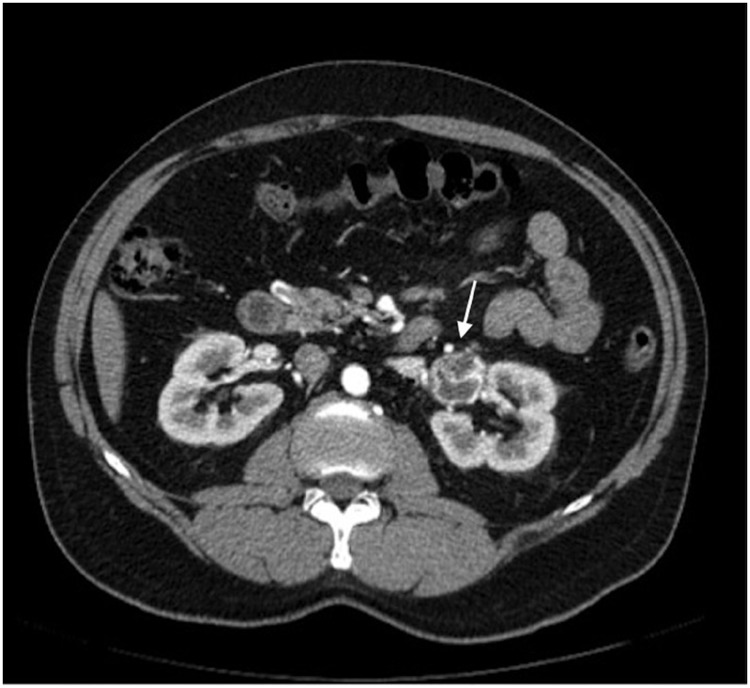
Corticomedullary phase from the urotomography demonstrates the lesion (arrow) with wall and septa enhancement.

A 53-year-old man underwent computed tomography (CT) for renal stone evaluation. His physical examination was otherwise unremarkable. His creatinine level was 1.0mg/dL and his fasting glucose was 91mg/dL. An incidental left renal mass was identified (Figure-1), that was further evaluated with magnetic resonance imaging (MRI).

**Figure 2:**
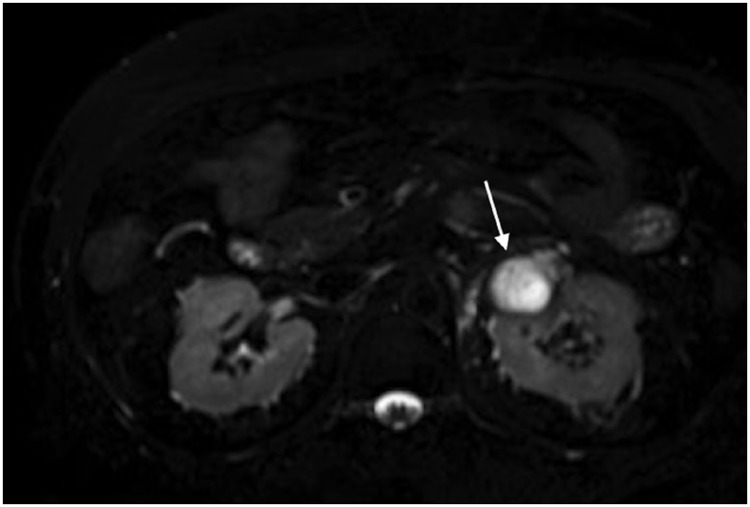
Axial T2 imaging with fat saturation shows an expansive, exophytic lobulated mass with high signal, (arrow) in the upper pole left kidney.

**Figure 3:**
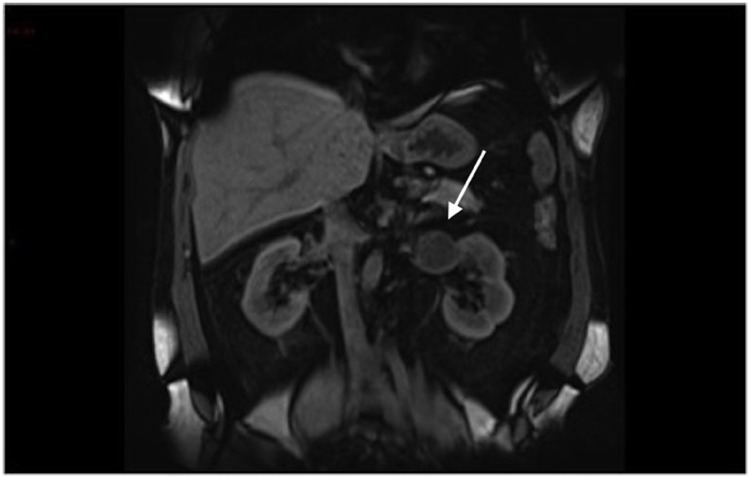
Coronal T1 pre-contrast imaging – the lesion is hypointense to adjacent renal parenchyma (arrow).

**Figure 4:**
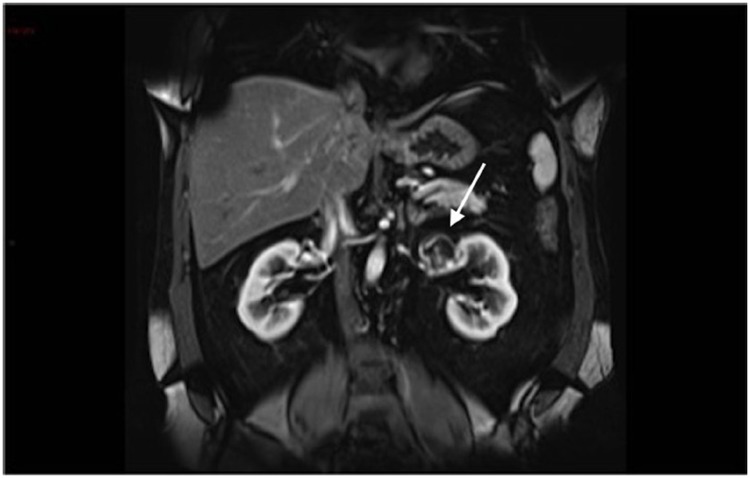
Coronal T1 post-contrast arterial phase imaging showing peripheral enhancement (arrow).

**Figure 5:**
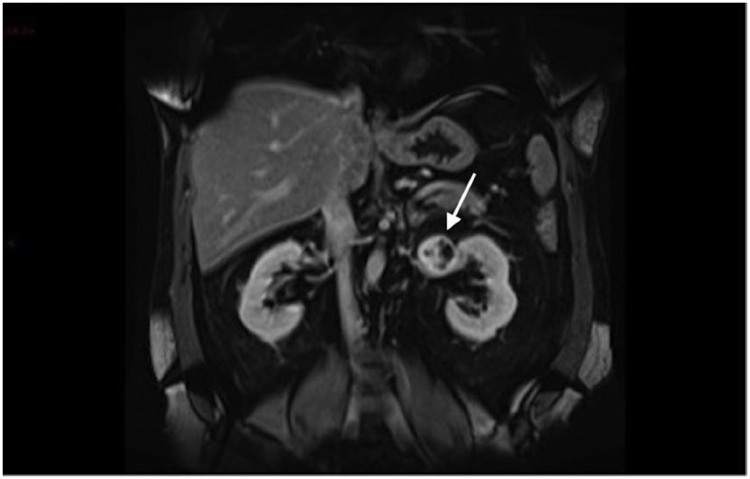
Coronal T1 late post-contrast phase shows progressive enhancement of the lesion.

MRI showed a renal mass with thick septa and progressive enhancement after gadolinium injection. The lesion was interpreted as a complex renal cystic lesion, classified as Bosniak IV (Figures 2–5).

**Figure 6:**
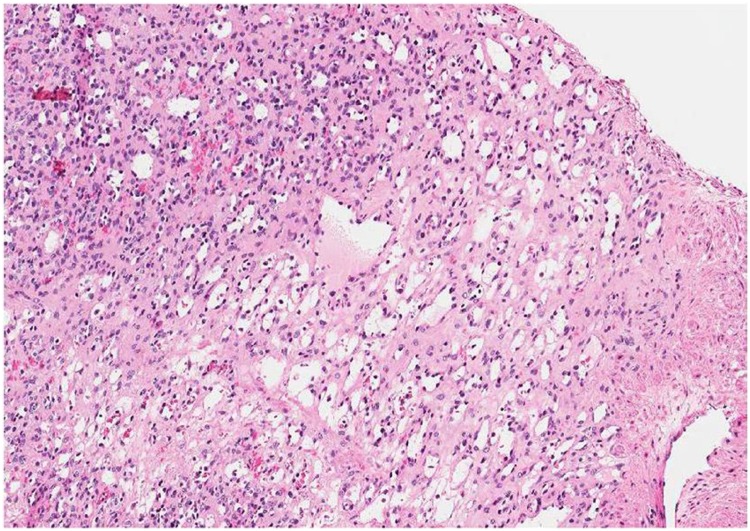
Histologic sample of the ressected lesion shows anastomosing proliferation of capillary sized vessels, reminiscent of splenic sinusoids and scattered hobnailed endothelial cells, confirming the diagnosis of an anastomosing hemangioma

After MRI results, patient underwent video-laparoscopic resection of the lesion, later confirmed to be a renal anastomosing hemangioma by histopathological analysis (Figure-6).

## DISCUSSION

Renal vascular tumors are extremely rare, with hemangiomas being the most frequent lesion in this subgroup (1).

The vast majority of renal hemangiomas are smaller than 2cm, asymptomatic and incidentally found on imaging exams. Symptomatic patients may have recurrent episodes of hematuria and abdominal pain (1, 2).

The anastomosing hemangioma is a rare variant, which histologically simulates an angiosarcoma (3). This histological subtype has been recently described as morphological variant of hemangioma that occurs primarily in the genitourinary tract. On non-enhanced CT, they are lobulated lesions, with soft-tissue attenuation. After contrast administration, they appear as solid heterogeneous lesions, with intense and progressive enhancement (3).

On MRI, hemangiomas show hyperintensity on T2 and variable degrees of enhancement after contrast administration. Presentations may resemble cystic lesions with solid component, mimicking cystic renal cell carcinoma as the present case (1, 2, 4). When large, these lesions are indistinguishable from malignant lesions such as angiosarcomas and renal cell carcinomas with central necrosis.

Treatment is controversial since preoperative diagnosis is not possible based on imaging exams. When biopsy results are available, it may vary from expectation to partial nephrectomy, embolization and radical nephrectomy, depending on the lesion size, location and presence of symptoms.
